# The dosimetric effect of photon target degradation on C‐series linear accelerators using Monte Carlo simulation

**DOI:** 10.1002/acm2.70208

**Published:** 2025-08-21

**Authors:** Yongjin Deng, Minmin Qiu, Jiajian Zhong, Zhenhua Xiao

**Affiliations:** ^1^ Department of Radiation Oncology The First Affiliated Hospital Sun Yat‐sen University Guangzhou China

**Keywords:** C‐series linear accelerator, Monte Carlo, off‐axis factor, relative output factor, target degradation, wedge factor

## Abstract

**Background:**

The impact of beam dosimetry changes brought on by target degradation has not been well investigated.

**Purpose:**

To determine the optimal dosimetry metrics for detecting target degradation and to investigate the impact of beam dosimetry changes brought on by target degradation.

**Methods:**

The EGSnrc Monte Carlo programs were used to model the Varian Novalis Tx linear accelerator. By altering the central portion of the photon target with a cylindrical vacuum zone, few target degradation scenarios were simulated and compared with the nominal condition. For square photon beams defined by the jaws, the effects of target degradation on beam output, beam quality (percentage depth dose, PDD), beam profile (off‐axis factor), relative output factor (ROF), and wedge factor (WF) were examined. Few degradation scenarios were used to retroactively model the effects on patient‐specific quality assurance (PSQA).

**Results:**

Under the realistic degradation conditions (0.6–0.7 mm depth, 1.0–2.0 mm widths), the 6MV beam output decreased by 5.7%–24.1% and the 30 cm field diagonal profile sag in the horns by a max deviation of 3.8%–5.9%, however, the PDD_20,10_ varied within 1%, and the flatness deviated by 0.74%–1.35%, respectively. As the photon target got more degraded, the beam output, beam quality and profile showed a downgrade trend from the baseline. The PDD_20,10_ varied from 0.7% to ‐2.8% in the 10 cm field. Before the target degraded with a 2 mm width and 0.889 mm depth hole, ROF and WF were stable (within 2% deviation). The pelvic case gamma result showed obvious decrease (90.9%) under degradation depth 0.7 mm and width 2.0 mm.

**Conclusion:**

A technical approach was created and verified for precise Monte Carlo simulation of linear accelerator target degradation scenarios. The parameters with the highest sensitivity for detecting target degradation were beam output and large field diagonal profile deviation in the 0.95 field size range.

## 1 INTRODUCTION

External beam radiotherapy (RT) frequently uses linear accelerators, also known as linacs, which accelerate electrons into a tungsten target to produce photon beams. Intensity‐modulated radiotherapy (IMRT) requires longer beam time that the Varian C‐Series linacs target is not designed for, hence increasing the likelihood of degradation.^1^ The beam steering system controls the beams focal spot position on the target,^2^ which is not necessarily stable during gantry rotation and machine initialization.^3^ The beam output, flatness, and symmetry will alter if the focus point crosses over into or enters the target defect region. This results in sub‐optimal treatment delivery. The Varian C‐Series Novalis Tx (NTX, Varian Medical Systems, Palo Alto, California, USA) linac in our department had faced target replacement after 4 years of operation. This machine primarily used the 6 MV photon beam for treatment and performed approximately 60–90 VMAT or IMRT plans per day. During the few weeks before the target replacement, we found the 6MV beam output decreased by 2% in one day and remained unstable after several beam output recalibrations. The beam output increased by 12.2% when measured immediately after the target was changed. The motivation of this study was to find out how this happened.

Few studies have focused on photon target degradation. When the photon target is degraded, it means that the target material at the focal spot melts, recrystallizes, and solidifies^4^ forming holes of varying depths. Typically, the target degrades very slowly and does not affect the machine's dose parameters for several years, but its degradation will eventually affect the dose at the end of its useful life. In tomotherapy, it has been shown to affect the dose to the prostate by 4.5%,^5^ and in C‐series linac, it has been shown to affect the beam output by 6.78%.^6^ Metrics such as 3% variation in diagonal normalized flatness^7^ and symmetry of the enhanced dynamic wedge field^8^ have been proposed to detect photon target degradation. However, these studies have not directly addressed the extent of target degradation and its relationship to the dose received by the patient.

The Monte Carlo particle transport technique models the energy deposition of physical particles in media and is often regarded as the most accurate dose calculation algorithm, including stopping ratios,^9^ correction factors,^10^ and absolute dose calculation.^11^ In this work, we apply it to target degradation simulations, we performed target structure modification during linac modeling, simulated target degradation conditions, and quantitatively investigated the effect of photon dosimetry, including beam output, relative output factor, wedge factor, PDDs, profiles in the diagonal, x, and y planes, and patient‐specific quality assurance (PSQA). To the best of our knowledge, no prior research has used the MC approach to simulate target degradation in medical linac. The method enables us to detect target degradation in real‐world applications, lessen its impact on clinical therapy, and investigate the influence of the target degradation process on radiation dose in patients early and more systematically.

## 2 MATERIALS AND METHODS

### 2.1 Monte Carlo simulation and commissioning

**FIGURE 1:**
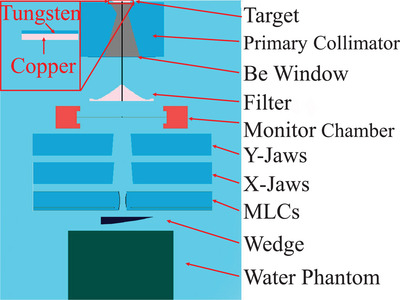
The main components of the simulated accelerator (not drawn to scale).

The radiation structure of the NTX accelerator was simulated using the BEAMnrc from the EGSnrc package.^12^ Model data were obtained from the Varian Monte Carlo Data Package (MCDP) provided by the accelerator manufacturer. The packaging was the same for the C‐Series high energy linacs NTX, Trilogy, CLINAC iX, DX, C/D, EX, and cX. The dose deposited in the monitor chamber was calculated during simulation and used for absolute dose correction. Figure 1 depicts the main components of the C‐Series linac. The first part is the photon target, which is made up of two layers. The 0.889 mm thick tungsten button is supported by a copper base that is 1.575 mm thick.

The DOSXYZnrc package was used to simulate 3D dose distribution in a 48×48×48 cm^3^ water phantom, and the PDDs and profiles were taken from output “3ddose” files. Source 9 Beam Treatment Head from BEAMnrc was used as input. This mode can save disk space from storing the phase space file and consume comparable simulation time. The global electron cutoff energy (ECUT) was set to 0.7 MeV, while the photon cutoff energy (PCUT) was set to 0.01 MeV. To increase simulation efficiency, directional bremsstrahlung splitting (DBS)^13^ was used, with a splitting number of 2000 and a configurable splitting radius [maximum diagonal distance at source to skin distance (SSD) of 100 cm].

The optimization method for the initial electron energy and a Gaussian intensity profile with full width at half maximum (FWHM) beam model parameters is based on the method suggested in literature.^14^, ^15^ To make the model feasible, the parameters were adjusted until they matched the measured PDDs and profiles for the static fields listed in Section 2.1.2.

All simulations used a single workstation with an Intel XEON E5 2620 2 GHz 12×2 processors CPU. For all data processing, analysis, and visualization were performed using in‐house MATLAB scripts (version 9.12.0 R2022a).

#### 2.1.1 Absolute dosimetry

The dose generated from the “3ddose” file has been normalized and should be converted to absolute dose. The calibration settings for this study were a 10 cm reference field at 100 cm SSD and maximum dose depth on the central axis (CAX) with $D_{\mathrm{Abs}}^{Cal}=1cGy/MU$. The absolute dose was based on the formula^11^ and rewritten to account for target degradation:

(1) $$\begin{array}{c} D_{Abs}^{f}\;\left(\frac{cGy}{per\;MU}\right)=D_{dpp}^{f}\;\times \frac{{D_{ch}}_{dpp}^{f,\;NC}}{{D_{ch}}_{dpp}^{f,Degradation}}\times \frac{{D_{ch}}_{dpp}^{10,NC}}{{D_{ch}}_{dpp}^{f,NC}}\\ \times \frac{D_{Abs}^{Cal}}{D_{dpp}^{Cal}}=D_{dpp}^{f}\;\times R_{ch}^{f,Degradation}\times F_{backscatter}^{f,NC}\times F_{cal} \end{array}$$

The absolute $D_{Abs}^{f}$ in a specific field *f* was obtained from dose results $D_{dpp}^{f}$ in DOSXYZnrc simulation with correction. The correction factor $R_{ch}^{f,\;Degradation}$ is the ratio of the dose in the monitor chamber under the nominal condition (NC) to the target degradation scenarios. If the target is under NC, the ratio will be a constant 1. $F_{backscatter}^{f,NC}$ is the backscatter correction factor under NC. The output of the modeled linac is affected by backscatter from the jaws to the monitor chamber, as the set number of MU is reached later as the field size increases due to less backscatter from these fields relative to the reference field. $F_{cal}$ represents the particle counts per MU of the calibration condition. This study's simulated calibration condition dose yielded $F_{cal}$ of 9.3925×10^15^ ± 0.24%.

#### 2.1.2 Static field commissioning

The static field commissioning compared the measured and simulated PDDs, profiles, and ROFs of static 6MV photon beams. All static photon fields in this study were square fields shaped by secondary collimators, and the field size was defined as the projection size at the SSD 100 cm position. Six open fields (3, 6, 10, 15, 20, and 30 cm) and three 30° wedge fields (10, 20, 30 cm) were simulated and utilized for commissioning. The PYRAMIDS module was used to model the wedge.

To generate a type A uncertainty of 1% or less for the voxel water phantom dose, simulated histories ranging from 1 to 8×10^9^ were selected. The grid size is 2×2×1 mm^3^, except for 6×6×1 mm^3^ (central part that avoid the penumbral region) when simulating the flat area in 30 cm field.

All simulated fields were measured using the CC13 ionization chamber (IBA dosimetry GmbH Germany) in the blue water phantom (IBA dosimetry GmbH Germany) at the same configuration as simulation. The measured results were interpolated into 1×1×1 mm^3^ grid. Following backscatter correction, the ROFs were contrasted with measurements from other study.^11^

#### 2.1.3 Patient‐specific quality assurance commissioning

The PSQA is an end‐to‐end patient plan validation procedure by comparing MC simulated dose planes with TPS calculated dose planes. The TPS calculated dose planes are validated using measurements in MatriXX. RT treatment plans were computed (Eclipse with AAA algorithm, version 13.0) and simulated in a MatriXX detector with a MULTICube phantom (IBA dosimetry GmbH Germany) in a 3×3×3 mm^3^ grid for a head and neck (H&N) cancer case using the RapidArc (Arc) technique and a breast cancer case using the sliding window (SW) technique, which were common cases treated in the linac.

**FIGURE 2:**
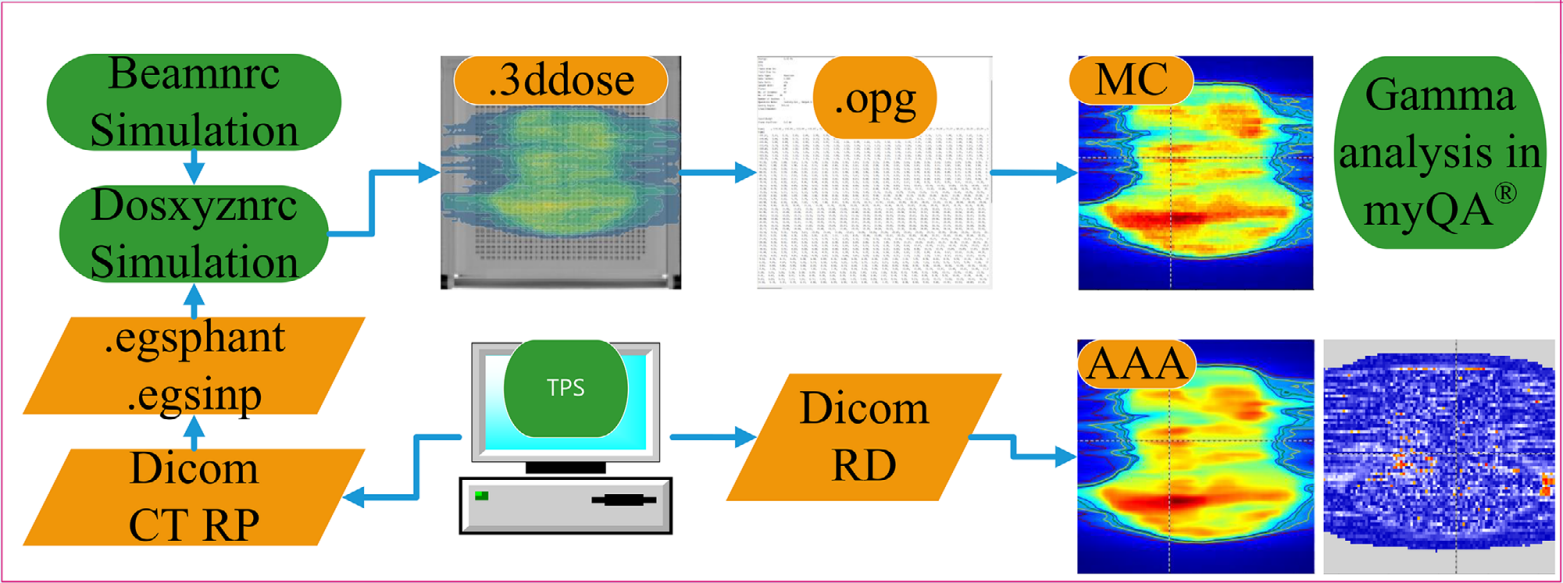
Patient‐specific quality assurance workflow.

The Eclipse DICOM (Digital Imaging and Communications in Medicine) RT Dose (RD) files and the DOSXYZnrc “3ddose” files (converted to “opg” format) were imported into myQA (IBA Dosimetry GmbH Germany) for absolute dose gamma analysis, as shown in Figure 2. The absolute dose conversion was based on the point dose conversion. The voxel dose was calculated by multiplying the number of MUs and the backscatter correction factor for each field simulation. The DICOM CT format phantom was converted using the real CT ramp to a DOSXYZnrc compliant “egsphant” format for plan recalculation. To reduce simulation time, the couch was removed from the phantom in both the MC simulation and the Eclipse TPS.

The multileaf collimator (MLC) was simulated using the SYNCHDMLC module, and certain settings were adjusted based on measurements made at McGill University in Quebec.^16^ A published script^17^ was used to extract the isocenter coordinate, MU, and collimator and MLC coordinates from the Eclipse‐exported DICOM RP files. These served as the BEAMnrc and DOSXYZnrc input settings. The in‐field dose uncertainty was less than 2% with 3×10^9^ simulation histories. Simulation time were roughly 7–13.5 h for the clinical Arc and SW plans with 178×2 and 79–104 subfields per treatment field, respectively.

### 2.2 Target degradation simulation

To assess the impact of target degradation scenarios on dosimetry in water, open and wedge fields were simulated in the same way (except for the target, and only 10, 20, and 30 cm fields) as the commissioning part mentioned in Section 2.1. The FLATFILT module was used to model the target structure and define a cylindrical vacuum zone in the center.

**FIGURE 3:**
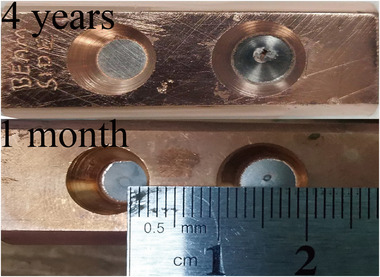
Degraded holes on 4‐year (top) and 1‐month (bottom) old targets on the NTX linear accelerator.

Figure 3 shows a 6MV photon target that is 4 years and only 1 month old, with apertures ranging from 1 to 2 mm. We designed the simulated targets with a starting width of 1 mm and different degradation depths and widths to mimic the real‐world target degradation process, and the simulated nominal target is designated NC. Table 1 provides more information on the degradation scenarios. To find the most realistic degradation condition, we fine‐tuned the depth and width in 0.2 mm increment before the degradation depth exceed the depth of the tungsten layer (0.889 mm), and named the degradation condition according to it, for example, we named the degradation condition with a depth of 0.6 mm and a width of 2.0 mm as D0.6W2.0.

**TABLE 1:** The setup examples of different target degradation scenarios.

Target degradation name	Depth of degradation (mm)	Width of degradation (mm)	Target degradation name	Depth of degradation (mm)	Width of degradation (mm)
NC	0	0	D0.7	0.7	1.0
D0.1	0.1	1.0	W1	0.889	1.0
D0.3	0.3	1.0	W2	0.889	2.0
D0.5	0.5	1.0	WC	1.889	2.0
D0.6	0.6	1.0	Z2	2.464	2.0
D0.6W2.0	0.6	2.0	Z3	2.464	3.0

*Note*: W1 and W2 represent the total degradation of the tungsten layer, with a width of 1  and 2 mm, respectively. WC represents the total degradation of the tungsten layer and the degradation of the upper 1 mm thick copper layer, with a width of 2 mm. Z2 and Z3 represent the total degradation of the two target layers, with a width of 2  and 3 mm, respectively.

### 2.3 Impact of target degradation on dosimetry

We calculated the beam output deviation at Z_10cm_ for different target degradation scenarios when compared with nominal target condition, because this depth is beyond the electron range and thus avoids electronic contamination is commonly used clinically. To evaluate the impact of target degradation on the ROF and WF, 20 and 30 cm fields were simulated at a few degradation scenarios, and the ROF equation was described below:

(2) $$ROF=\frac{D_{Abs}^{f}}{D_{Abs}^{10}}=\frac{D_{dpp}^{f}}{D_{dpp}^{10}}\;\times \;\frac{{D_{ch}}_{dpp}^{10}}{{D_{ch}}_{dpp}^{f}}=\frac{D_{dpp}^{f}}{D_{dpp}^{10}}\;\times F_{backscatter}^{f}$$

We found the factor $F_{backscatter}$ under target degradation scenarios differed from that at NC conditions by less than 1%. To reduce error propagation, the $F_{backscatter}$ under target degradation conditions were set the same under the NC.

The WF was calculated for NC and W1 conditions:

(3) $$WF=D_{Abs}^{WG}/D_{Abs}^{open}=\frac{D_{dpp}^{WG}}{D_{dpp}^{open}}\times \;\frac{{D_{ch}}_{dpp}^{open}}{{D_{ch}}_{dpp}^{WG}}=\frac{D_{dpp}^{WG}}{D_{dpp}^{open}}\;$$

$D_{Abs}^{WG}$ was simulated dose at 100 cm SSD and Z_max_ on the CAX with the wedge in the beam, whereas $D_{Abs}^{open}$ was dose at the same conditions without the wedge. The ratio $\frac{{D_{ch}}_{dpp}^{open}}{{D_{ch}}_{dpp}^{WG}}$ was set to constant 1, because the wedge was positioned far away from the monitor chamber to have little effect on its response.

Statistical changes in PDD, profiles, PDD_20,10_, and flatness^18^ were investigated for 10, 20, and 30 cm fields under several target degradation scenarios. The PDD_20,10_ is the ratio of PDD at Z_20cm_ and Z_10cm_ (depths of 20 and 10 cm) for a 10 cm field at 100 cm SSD and can be regarded as a beam quality parameter.^19^ The American Association of Physicists in Medicine (AAPM) task group 142 report^20^ suggested the PDDs and profiles (at any position within 0.8 field size) should be within a relative change of 1% from the baseline measured with a QA device immediately following beam commissioning or updated by the annual review. The simulated PDDs and profiles data were processed using a second‐order adaptive Savitzy‐Golay filter to better quantify changes under target degradation. In this work, we used the simulated target NC condition as the linac's baseline state.

The maximum beam profile deviations $Deviation_{max}$ from baseline were calculated as follows:

(4) $$\;Deviation_{max}=MAX\left({\left|\frac{TP_{L}-BP_{L}}{BP_{L}}\right|}_{L\;in\;OAFs}\times 100\%\right)$$

$TP_{L}$ is the test point L in beam profile, $BP_{L}$ is the same point in baseline situation.

We collect two $\mathrm{Deviatio}n_{\max}$ data at one beam profile curve, one from the negative axis region and one from the positive axis region. The $\mathrm{Deviatio}n_{\max}$ for OAFs of open or 30° wedge field along diagonal/x/y axes at 5 cm depth (Z_5cm_) and 10 cm depth (Z_10cm_) were collected and analyzed, and the median, mean and standard deviations of $\mathrm{Deviatio}n_{\max}$ in the 10, 20, and 30 cm fields were calculated. Friedman's test was performed included all field sizes, depths and axis directions to determine whether there were significant differences in the $\mathrm{Deviatio}n_{\max}$ caused by the interest axes (diagonal and x/y axes), interest areas (central 0.8 and 0.95 field size), wedge, and depths. Given the existence of systematic errors in the Monte Carlo simulation, the more obvious data for the W1 case were chosen for testing.

The flatness of 10, 20, and 30 cm fields under NC, W1, and W2 conditions were calculated at Z_10cm_ and 100 cm SSD as follows:

(5) $$Flatness=\frac{M-m}{M+m}\;\times 100\%$$

M is the maximum and m is the minimum value in the central 0.8 field size of profiles along the diagonal and x/y beam axes.

### 2.4 Impact of target degradation on patient‐specific quality assurance

To assess the impact of target degradation on PSQA, 2D gamma analysis of a H&N case and a pelvic case were performed to compare with NC targets in a few target scenarios, especially in width 2.0 cm for its larger beam profile deviation in static field. In the pelvic case, the arc field that cover the inferior part of the PTV target was chosen, because the HD120 MLC of the NTX linac was limited to 22 cm in the Y axis, but the pelvic target area exceeded ‐13 cm in the Y axis, this field was rotated 90° to allow the MLC projection to cover the treatment area. The analysis is Monte Carlo of the target degradation cases compared to Monte Carlo of the non‐degradation case. The isocenter dose under target degradation was rescaled to that at NC target condition. This was in line with the real world, because even if the target was degraded, daily morning checks and recalibrations guaranteed the beam output to be within ± 2% of the baseline output.

## 3 RESULTS

### 3.1 Benchmarking of the simulated linac

#### 3.1.1 Comparison of PDDs and profiles with measured results

With the setting of initial electron beam energy of 5.7 MeV and a Gaussian FWHM of 0.1 cm, the difference between the simulated and the CC13 measured PDDs and profiles was less than 1% in every static field case except for the peak area of the profile of the 30° 30 cm wedge field. Details can be seen in the Supplementary figures. It is reasonable to believe that the precision of the model matches the measurement and that it can be applied to target degradation research.

#### 3.1.2 Comparison of ROF with measured results

The simulated ROF values were compared with the results simulated and measured in other studies using a 21EX accelerator.^11^ The difference was ‐0.5% to 0.5%. Based on the consistency model between 21EX and NTX, it can be concluded that the MC simulation in this work is reliable.

#### 3.1.3 Patient‐specific quality assurance experiment commissioning

The plan field dose uncertainties deposited in the monitor chamber were within 0.5%, and all patient plans for Eclipse that were previously tested using the MatriXX phantom met the suggested gamma requirement of 3%/2 mm > 95%.^21^ As shown in Table 2, our Monte Carlo simulation of the coronal planar dose agreed with the calculation result in Eclipse. After combining the individual fields, the Arc plan had a 2%/2 mm 96.5% pass rate, and the SW plan had a 2%/2 mm 92.7% pass rate.

**TABLE 2:** Gamma verification of the MC simulated dose and the Eclipse calculated dose in a MatriXX phantom, at isocenter coronal plane, for a clinical H&N Arc plan and a breast SW plan.

	Arc Field 1	Arc Field 2	SW Field 1	SW Field 2	SW Field 3	SW Field 4	SW Field 5
F_backscatter_ ^a^	1.0137	1.0075	1.0032	1.0034	1.0029	1.0082	1.0110
Gamma pass rate(%)^b^	97.8	96.8	96.2	98.4	98.7	96.5	96.9

Abbreviations: Arc, RapidArc; Fbackscatter = the collimator backscatter correction factors; SW, sliding window.

^a^ The Fbackscatter were calculated from simulated data using Equation (1).

^b^ Criteria of 2%/2 mm.

### 3.2 Impact of target degradation on beam output, relative output factor, wedge factor

The beam output continued to decrease as the degradation depth increased, decreasing by 97.1% for the WC target, but returning to approximately 50% of baseline for the Z2 and Z3 targets. The chamber response ratio had the greatest effect on output. Table 3 listed the chamber response ratio $R_{ch}^{Degradation}$ at 10 degradation scenarios. There was 5.8% more dose deposited in the monitor chamber under target D0.6 and 17.2% more under target D0.7 when compared to target NC.

**TABLE 3:** The 10×10 cm^2^ $R_{ch\;}^{10,Degradation}$ ratio for 10 target degradation scenarios.

Target degradation scenarios	$R_{ch\;}^{10,Degradation}$ with MC unc.	Target degradation scenarios	$R_{ch\;}^{10,\;Degradation}$ with MC unc.
D0.1	1.003 ± 0.012	W1	0.453 ± 0.004
D0.3	0.984 ± 0.012	W2	0.312 ± 0.002
D0.5	0.982 ± 0.012	WC	0.031 ± 0.0002
D0.6	0.945 ± 0.007	Z2	0.394 ± 0.004
D0.7	0.853 ± 0.010	Z3	0.430 ± 0.004

Abbreviations: The chamber response ratio $R_{ch\;}^{10,Degradation}$ was the ratio of the dose in the monitor chamber under nominal condition to the target degradation scenarios. MC unc., standard deviation based on the 3ddose results.

**FIGURE 4:**
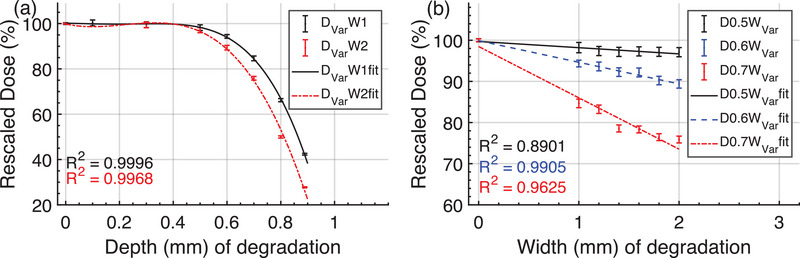
(a) Effect of variable degradation depths on beam output for degradation widths of 1  and 2 mm (D_Var_W1, D_Var_W2) and (b) effect of variable degradation widths on beam output for depths of 0.5, 0.6, 0.7 mm (D0.5W_Var_, D0.6W_Var_, D0.7W_Var_). The scatters with ± SD error bars are absolute dose calculated over the 10 cm field at Z_10cm_ depth in the water phantom and normalized to NC case. The curves are polynomial fits to the scatter points.

Starting at a degradation depth of 0.6 mm, the output varied sharply with degradation depth, but relatively flatly (especially at depths of 0.5 and 0.6 mm) with degradation width as shown in Figure 4. The beam output varied within 1% of the baseline under degradation depth 0.1–0.3 mm, and it decreased by 1.8%–2.9% (D0.5W_Var_), 5.7%–10.6% (D0.6W_Var_), and 15.4%–24.1% (D0.7W_Var_), respectively. For the simulated degraded depth of 0.6–0.7 mm, width 1.0–2.0 mm, the beam output varied not far from ‐6.78%^6^ and ‐12.2 % output changes reported for real‐world target degradation instances in the Introduction section.

**FIGURE 5:**
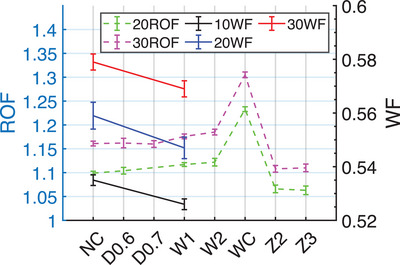
The impact of target degradation on ROF (at Z_10cm_) and WF (at Z_max_) with a ± SD error bar, on CAX for 10 , 20 , and 30 cm fields at 100 cm SSD.

As shown in Figure 5, the ROF gradually increased as the target was penetrated deeper. Under target WC, it increased dramatically, reaching 12.2% for the 20×20 cm^2^. For degradation before target W2 condition, the change was within 2%. The ROF decreased at Z2 and Z3 conditions. All the 30° physical WF under target condition W1 were changed within 2% from baseline.

### 3.3 Impact of target degradation on PDD and profiles

**FIGURE 6:**
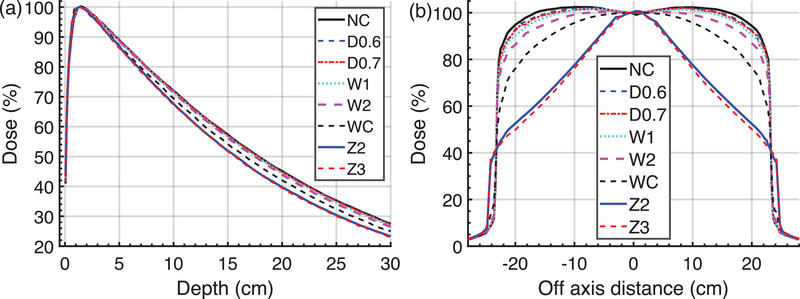
Impact of target degradation on (a) the PDD and (b) the Diagonal‐axis profile for the 30 cm field at Z_10cm_ depth.

The simulation results presented in Figure 6 indicate that the PDD and profile curves exhibited a measurable change as the photon target degraded. A significant Diagonal profile deviation was observed in the case of target W1. The profiles were stretched and rounded under target Z2 and Z3 conditions.

**FIGURE 7:**
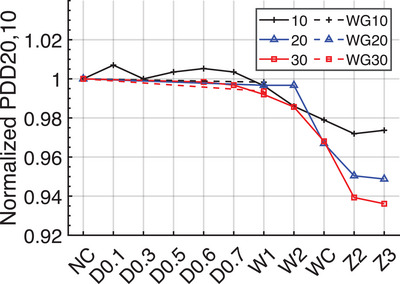
The impart of target degradation on PDD_20,10_ of the 10, 20, and 30 cm open (solid lines) and wedge fields (dashed lines), the connection lines were for demonstration purposes only, the results were normalized to NC case.

The PDD 20,10 was not sensitive to early degradation before W2. Under the same degradation depth of 0.6  and 0.7 mm, both 10 and 30 cm PDD 20, 10 varied within 1% when the width of degradation changed from 1.0 to 2.0 mm with 0.2 increments. As shown in Figure 7, under targets D0.1 to W1, the PDD_20,10_ for 10, 20, and 30 cm fields varied within 1% of the baseline (NC), while when the target was further degraded (W2), the PDD_20,10_ decreased by more than 1%. For 10 cm field, the range was 0.7% ∼ ‐2.8%, for 20 cm field, ‐0.3% ∼ ‐5.1%, and for 30 cm field, ‐0.2% ∼ ‐6.4%.

**FIGURE 8:**
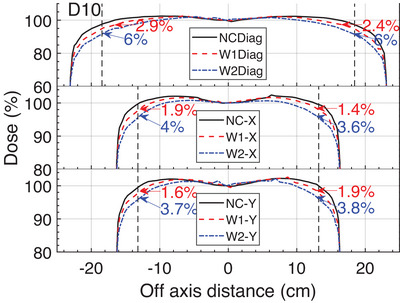
The example of quantified maximum deviations of 30 cm open‐field profiles for the target conditions of W1 and W2 from the baseline (NC) along the diagonal (Diag) and x‐y axes at 100 cm SSD and at Z_10cm_ (D10). The vertical dashed line indicates central 0.8 field size.

**FIGURE 9:**
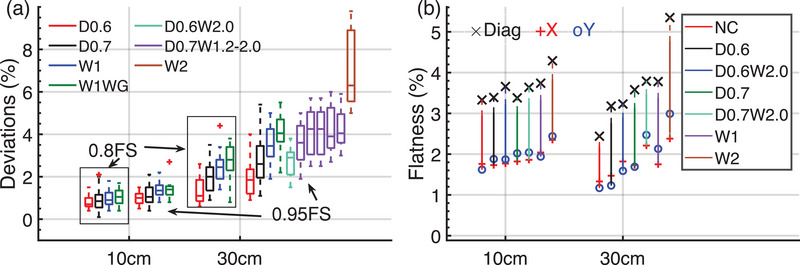
The impact of target degradation on (a) the maximum profile deviations from baseline, boxplot data were collected in both negative and positive side along the interest axes of 10 and 30 cm fields, W1WG means wedge field at W1 condition, D0.7 W1.2∼2.0 means 0.2 mm increment of width under 0.7 mm depth of degradation, and (b) flatness in diagonal, x‐y axes of 10 and 30 cm fields, the colored lines are to distinguish target conditions.

As shown in Figure 8, the maximum deviations were quantified. The diagonal profiles were more significant among the profiles in the x/y axes. Figure 9a shows that as the degradation depth increased, the profile deviation also increased, the profile deviation of 30 cm field was more sensitive than that of 10 cm field. The observed deviations in 0.95 field size central profiles varied from 0.4% to 2.7% for the field of 10 cm, and from 0.9% to 5.5% for the field of 30 cm under D0.6 D0.7 and W1 conditions. The median profile deviation at the target D0.6 began to exceed 1% (with a range of 0.6%∼2.6%) at 30 cm field, and when the region of interest was set to 0.95 field size, the median profile deviation was 1.85% (0.9%∼4%). Coincidentally, as the degradation width increased, the profile deviation also increased. The median profile deviation increased from 1.85% to 2.9% at the degradation depth of 0.6 mm, 2.6% (D0.7W1.0) to 4.05% (D0.7W2.0) at depth of 0.7 mm, but at the depth of 0.889 mm, the deviation increased from 3.45% (W1) to 6.3% (W2). The median profile deviations for wedge field were larger than that for open field under target W1 condition.

Figure 9b shows that as the target degraded, the flatness of the 30 cm field was more sensitive than that of the 10 cm field. The diagonal axis flatness showed an increase of 0.74%–0.79%under target D0.6, 1.14%–1.35% under target D0.7, and 2.91% under target W2, but the x and y axes flatness increased by only > 1% under target W2 and D0.7W2.0. The 10 cm flatness deviations were all within 1% from the baseline as the target degraded.

Table 4 showed that there was a significant difference (*p* < 0.05) in interest axes and interest areas groups, however, there was no significant difference in wedge and depths groups. The mean of Deviation_max_ was greater at 5 cm than at 10 cm in open field and reversed in wedge field.

**TABLE 4:** Impact of interest axes, interest areas, wedges, and depths on Deviation_max_ across all field sizes, depths, and axis directions under target degradation W1 condition.

Groups	Mean of Deviation_max_ (%)	SD (%)	*p*
Diag axis	2.9	1.2	<0.05^*^
X axis	2.2	1.1	
0.8FS	2.1	1.0	<0.05^*^
0.95FS	3.1	1.2	
Open	2.6	1.3	0.68
Wedge	2.5	1.2	
Z_5cm_ open	2.5	1.2	0.13
Z_10cm_ open	2.2	1.0	
Z_5cm_ wedge	2.4	1.1	0.06
Z_10cm_ wedge	2.9	1.4	

*Notes*: Deviationmax, maximum profile deviation from baseline. Interest axes = diagonal (Diag) and x axes. Z5cm = 5 cm depth, Z10cm = 10 cm depth. Interest areas = 0.8FS and 0.95FS, which were the central 0.8 and 0.95 field size range of profiles. Open = open fields. Wedge = 30° wedge fields.

Abbreviation: SD, standard deviation.

* Significant difference.

### 3.4 Impact of target degradation on patient‐specific quality assurance

**FIGURE 10:**
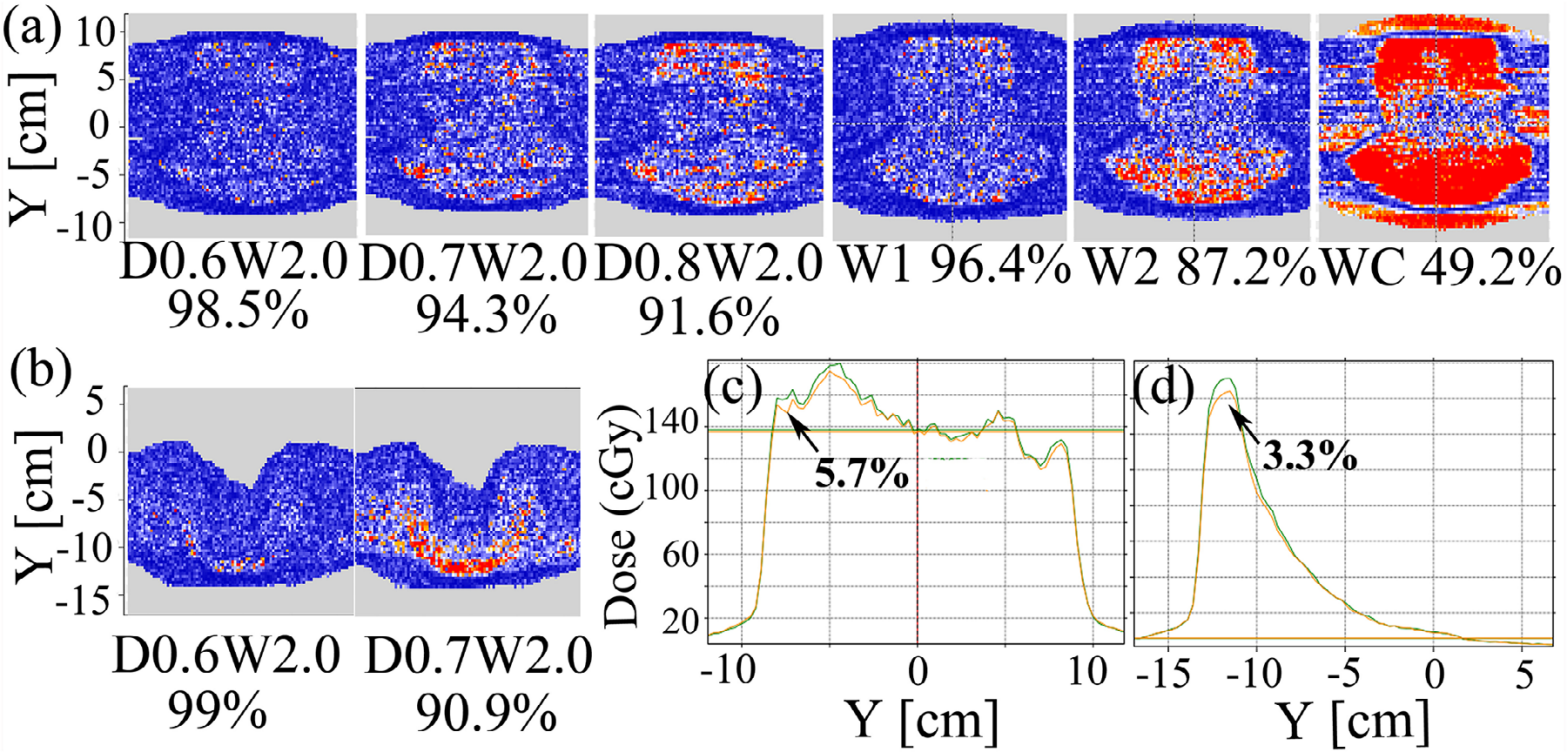
Impact of target degradation on PSQA. Top row (a) were H&N case and Bottom row (b) were pelvic case gamma index maps for the MC simulated target degradation distribution against that under nominal condition (NC). (c) The H&N case and the (d) Pelvic case dose profiles on the central axis in the y direction, green line was NC, and yellow line was the W2 and D0.7W2.0 target degradation condition, respectively.

PSQA of a H&N case and a pelvic case in the coronal plane are shown in Figure 10. The common H&N case treated in the linac passed the test at the 2%/2 mm > 95% criterion at D0.6W2.0 and W1. Compared with W1 (0.889 mm), D0.8W2.0 had a smaller degradation depth (0.8 mm), but a larger degradation width, and its gamma result was worse. The result for targets W2 and WC were 87.2% and 49.2%, respectively. The Pelvic arc field pass rate was 90.9% at D0.7W2.0. Major fails were in the periphery area of the Arc field with a maximum relative dose reduction of 5.7% (H&N W2) and 3.3% (pelvic D0.7W2.0) on the central axis in the y direction.

## 4 DISCUSSION

The incident electron energy of 5.7 MeV and a Gaussian intensity profile with a FWHM of 0.1 cm were optimal for matching the measurements. While the 5.9 MeV and 0.2 cm parameters were more suitable for larger field penumbra areas. However, the PDD and profile penumbra area deviations were not optimal for smaller fields. Another study has reported using 5.8 MeV and 0.12 cm FWHM.^22^ A notable unsolved discrepancy was observed between the measured and modeled profiles of the beam horns, particularly in the large wedge field. Such discrepancies can be observed in other studies.^23^, ^24^ It is recommended to verify the wedge parameters with real measurements, the same as HDMLC.^16^ Our simulated absolute doses in the clinical plan were slightly lower than Eclipse, which may be due to the systematic error of MC simulation and the presence of artifacts.^25^ Further interest will be taken in the leaf tip gap^26^ (since there were no modified MLC positions in this study) that may influence the simulated dose.

Similar output changes were recorded for simulated degraded target depth of 0.6–0.7 mm, width 1.0–2.0 mm to real‐world target degradation presented in the literature.^6^ This would suggest that those target simulations may be most representative of real‐world target failures. At this time, the CSSA software will issue a warning or interlock for 4DTC computer. The W1 to WC conditions are unlikely to occur due to the output drop being already of sufficient concern to replace the photon target. However, in extreme cases, that may also occur if the output was adjusted in the same direction for a long time and not enough attention was paid to the changes in the beam profile. The $R_{ch\;}^{WC}$ listed in Table 5 for 10 cm field is 0.031, which means the dose deposited on the monitor chamber under target WC is 32 times that at baseline status. This maybe because many primary electrons escape from the remaining 0.5 mm thick copper (which can be regarded as a thin target) and generate many unfiltered low‐energy photons. The Compton scattered photon and recoil electron will deposit more dose on the monitoring chamber. This does not affect the accuracy of our absolute dose simulations, the relative dose in 3ddose file has been converted to consider the calibration of the machine's monitoring chamber. The main difference for the Z2, Z3 is the direct interaction of the primary electron beam and the flattening filter, there is no photon produced at the photon target through the filter and deposited at the monitor chamber. It is also plausible that the shape of the filter would spread the photon distribution and absorb the scattered electrons. This results in less dose being deposited at the monitor chamber and returning to the output.

Anecdotally, an effective diagnostic method for detecting target degradation has been whether a difference between the 6 MV output and higher photon beam output is observed. However, for machines with only one photon energy mode, this cannot be checked. Our results show that there is no significant change in output from NCs to D0.5, but that the output drops by 3.9% from D0.5 to D0.6 and 9.7% from D0.6 to D0.7, the output starts to decrease increasingly for 0.1 mm thinning of the tungsten layer. The beam output and profile deviation were more sensitive than PDD_20,10_, flatness, ROF, and WF. Under the target condition D0.6, the diagonal profile deviation was up to 4%, while the changes of PDD_20,10_, and flatness were within 1%, and the changes of ROF and WF were within 2%. Combined with the safety notice of sudden changes in beam output of more than 3% in a single day and more than 6% in a single week,^1^ we can assume that when the target was already degraded, the tungsten layer thickness can change by 0.1 mm in a short period of time because a thinner tungsten layer is less able to dissipate heat. A 6% drop in output within a few weeks and further examination of > 3% diagonal deviation of the profile from baseline within the 0.95 field size region of interest (field of 30 cm or larger) are recommended metrics for detecting target degradation. Since the target degradation is a long‐term process that can get more degraded to affect the photon dosimetry in a few weeks, early target degradation can be found by regularly using a 3D water phantom or a 2D detector for large field diagonal beam profile scanning. If an > 1% profile deviation from the baseline at any field and axis was found during a monthly or annual QA check, additional action should be performed, for it could have major repercussions for patient.^27^

According to the quantitative analysis of the Monte Carlo simulated profiles of the various target degradation conditions, diagonal direction profiles at the middle 0.95 field size area were more sensitive because the region of interest was relatively wider. There were no appreciable deviations in the beam horns when comparing the profile of 30° wedge fields with those of the open fields. This could be a result of the relative changes in the low dose range being even more significant than the changes inside the wedge profile's peak dose range. Open fields profile deviations were more sensitive at Z_5cm_ than Z_10cm_, however, the wedge fields showed the exact opposite pattern, which might be attributed to the physical wedge plate making the beam harder.

All beam profiles in a degraded condition show flatter profiles at Z_5cm_ than those at baseline. The change in profile shows a decrease in horns, which means an increase in beam energy theoretically,^28^ but the PDD shows a decrease in beam energy. In theory, a thin target will soften the beam quality. This may be related to the fact that the x‐ray distribution is a convolution of the angular distribution of the electrons (when they interact) and the angular generation cross‐section, and more electrons directed forward near CAX because of target degradation means more x‐rays directed forward, resulting in relative reduced dose deposition at the edge of the central field compared to CAX. This phenomenon is not caused by the change in incident electron energy but by the degradation or melting of the target material, which is consistent with the results of measurements in the literature.^7^ Although the AAPM Task Group 142 report recommends a limit of ± 2% change in ROF, WF, and 1% change in PDD_10_ or TMR_20,10_.^20^ Our work suggests that a 1% change in these parameters indicates that target degradation is no longer negligible, because they should tend to be relatively stable. Gao et al.^7^ reported a 3% variation in diagonal flatness as an early warning sign of target degradation based on routine QA devices. Whereas in this study, only 1.14% variation in 30 cm diagonal flatness was observed in the D0.7 target condition and less than 3% diagonal flatness variation in the target W2 condition. This may be because the unstable symmetry was not considered in this work, and if the symmetry of the linac was the best state by default, then the flatness value seems more stable.

The lower gamma pass rate at the peripheral area within the treatment range of the same plan may help us detect target degradation, but it may not be sensitive in plans with small beam sizes. In the H&N case, the gamma results only showed an obvious deviation under target condition D0.8W2.0, but for slightly larger beam size of the pelvic case the pass rate decreased obviously under D0.7W2.0. Based on the limitation of 22 cm size in Y direction of HD120 MLC, we cannot select a more realistic clinical case with a beam size of 40 cm in length (such as metastatic lymph node lesions or multiple metastases), but it is foreseeable that cases with a larger irradiation field in common Varian C‐series linacs can better detect the phenomenon of reduced pass rate in areas away from the central axis under early target degradation D0.6.

The conclusions of this study are limited to Varian C‐series high energy linacs, which are being phased out and increasingly replaced by new TrueBeam‐type machines. Nonetheless, they still play an important clinical role outside Western countries. The TrueBeam “low‐X” target shared by the 6MV flat (6X) and flattening filter free (6FFF) beams is expected to show less target degradation after it was redesigned to increase the heat sink area to face higher MU. However, its material composition is the same as that of the C‐series machines. The 6FFF mode beam output appears to be more susceptible to target degradation than the 6X mode.^29^ The findings in this study, such as beam output and large field diagonal profile deviations from baseline, can still be used as metrics to detect target degradation in TrueBeam machines, especially the 6FFF beam, but further investigation is needed.

## 5 CONCLUSION

A technical method was used to simulate photon target degradation conditions for Varian C‐series high energy linacs and found that beam output and diagonal profile can be the most sensitive metrics of target degradation. A 6% decrease in output in a few weeks, a 3% deviation in large field diagonal profile, and ROF, PDD_20,10_, WF with only 1% of the change should be investigated further. Overall, the Monte Carlo model developed in this study performs well when compared to a commercial TPS and measurements in a water phantom, and it is a useful tool for investigating anomalies in the beam properties generated by a degrading linac target and developing second checks for dose verification.

## AUTHOR CONTRIBUTIONS

Yongjin Deng and Minmin Qiu contributed equally to this work.

## CONFLICT OF INTEREST STATEMENT

The authors have no relevant conflicts of interest to disclose.

## Supporting information



Supporting Information
