# Supplementary material for: The dosimetric effect of photon target degradation on C‐series linear accelerators using Monte Carlo simulation
Source: J Appl Clin Med Phys. 2025 Aug 21;26(9):e70208. doi: 10.1002/acm2.70208 (PMC12370408; doi:10.1002/acm2.70208)
Supplement: Supplementary file 1 — Supporting Information [file ACM2-26-e70208-s001.docx]

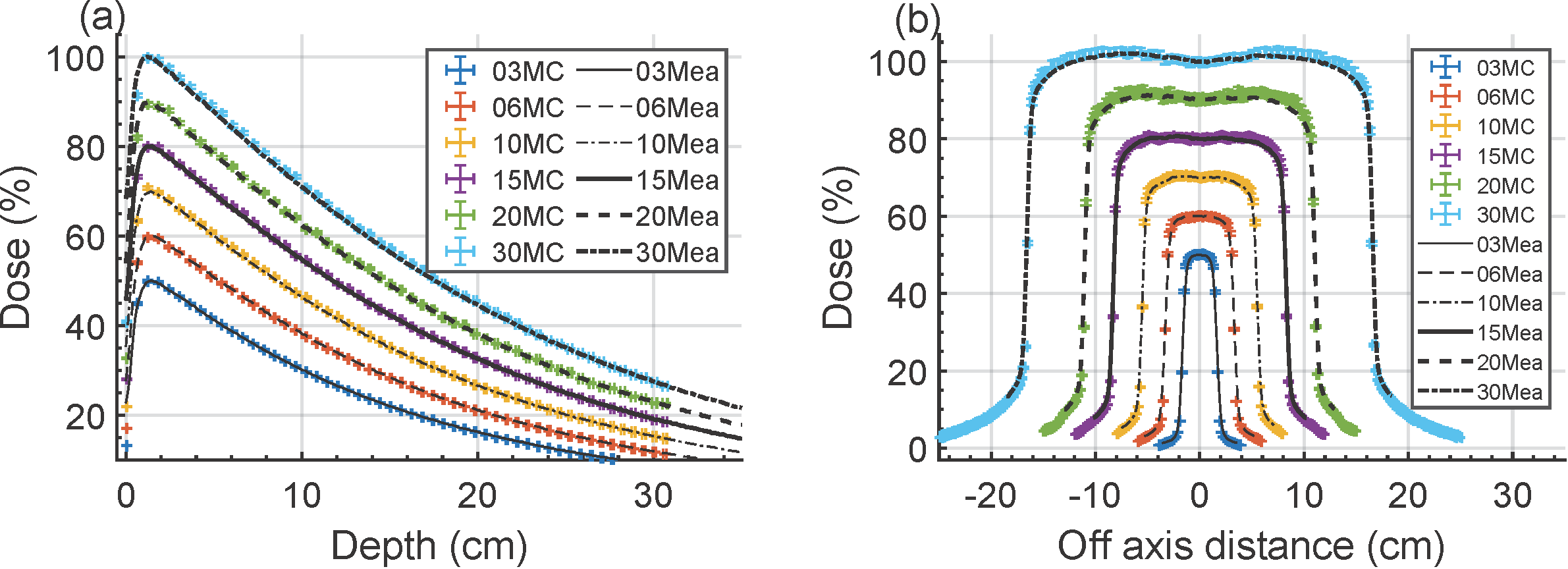


FIG. 1. (a) The simulated (with 0.5% error bar in x-y direction) and the measured (solid line) PDDs curves. (b) The simulated (with 1% error bar in x-y direction) and the measured (solid line) crossline (x plane) profiles at 10 cm depth for 3×3, 6×6, 10×10, 15×15, 20×20, and 30×30 cm^2^ open fields.


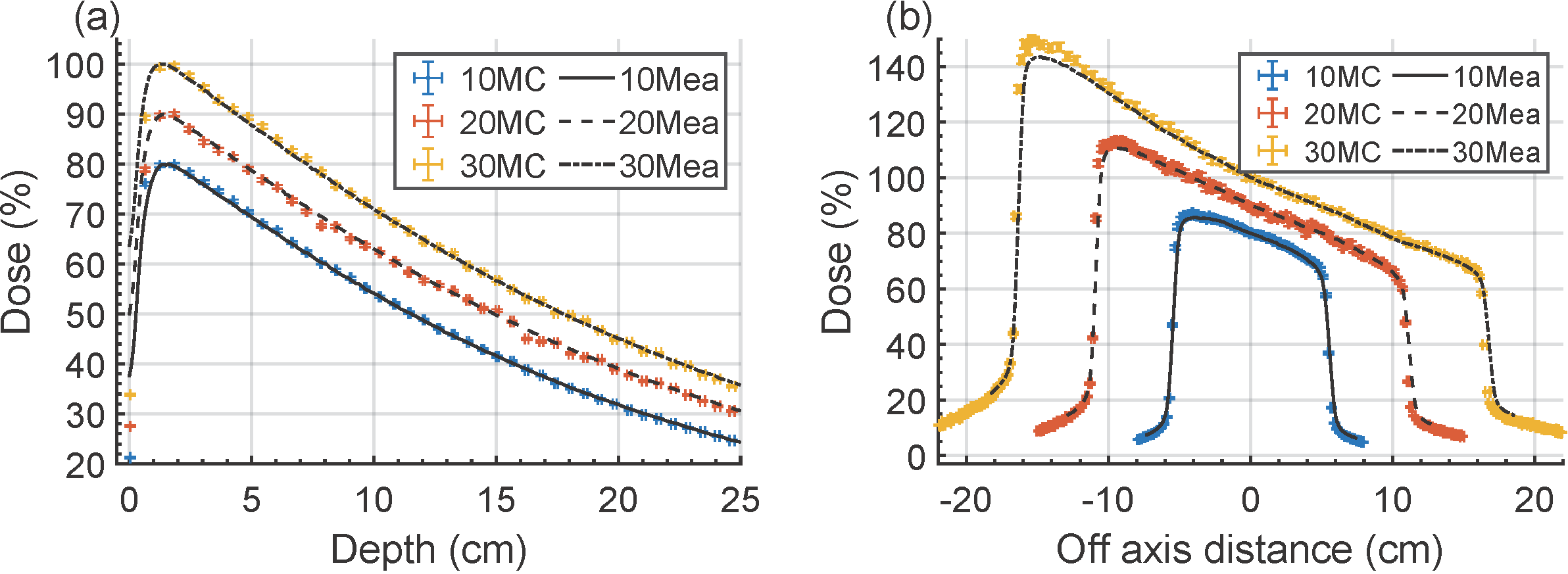


FIG. 2. (a) The simulated (with a 0.5% error bar in the x-y direction) and the measured PDDs (solid line), (b) the simulated (with 1% error bar in the x-y direction) and the measured (solid line) crossline (x plane) profiles at 10 cm depth for 10×10, 20×20, and 30×30 cm 30° physical wedge fields.

In FIG. 1, the maximum points of PDDs and the central points of profiles in 3×3, 6×6, 10×10, 15×15, 20×20, and 30×30 cm^2^ fields were rescaled to 50, 60, 70, 80, 90, and 100%, respectively. In FIG. 2 maximum points of PDDs and the central points of profiles were rescaled to 80% (10 cm), 90% (20 cm), and 100% (30 cm), respectively.
